# Supplementary figures and images for: Transition into inflammatory cancer-associated adipocytes in breast cancer microenvironment requires microRNA regulatory mechanism
Source: PLoS One. 2017 Mar 23;12(3):e0174126. doi: 10.1371/journal.pone.0174126 (PMC5363867; doi:10.1371/journal.pone.0174126)

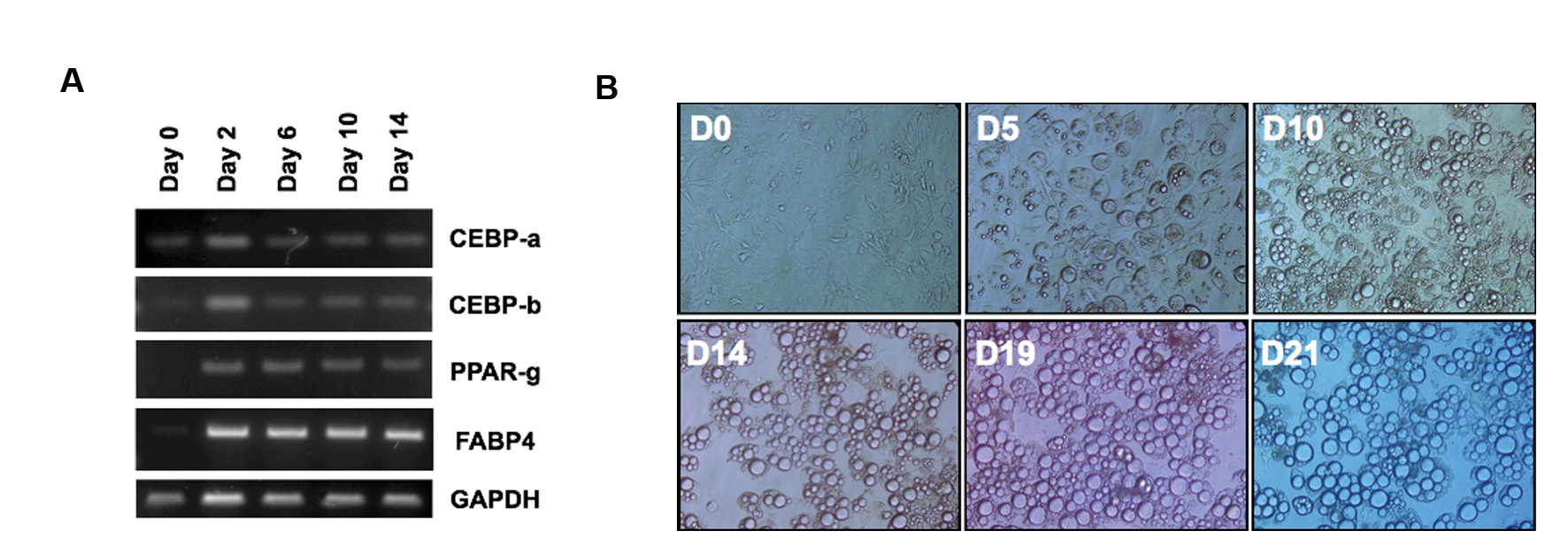

Supplement: S1 Fig — Along with the adipogenic gene expression (A), the cells showed increased accumulation of lipid drops and increase in the cell sizes (B). (TIF) [file pone.0174126.s001.tif]

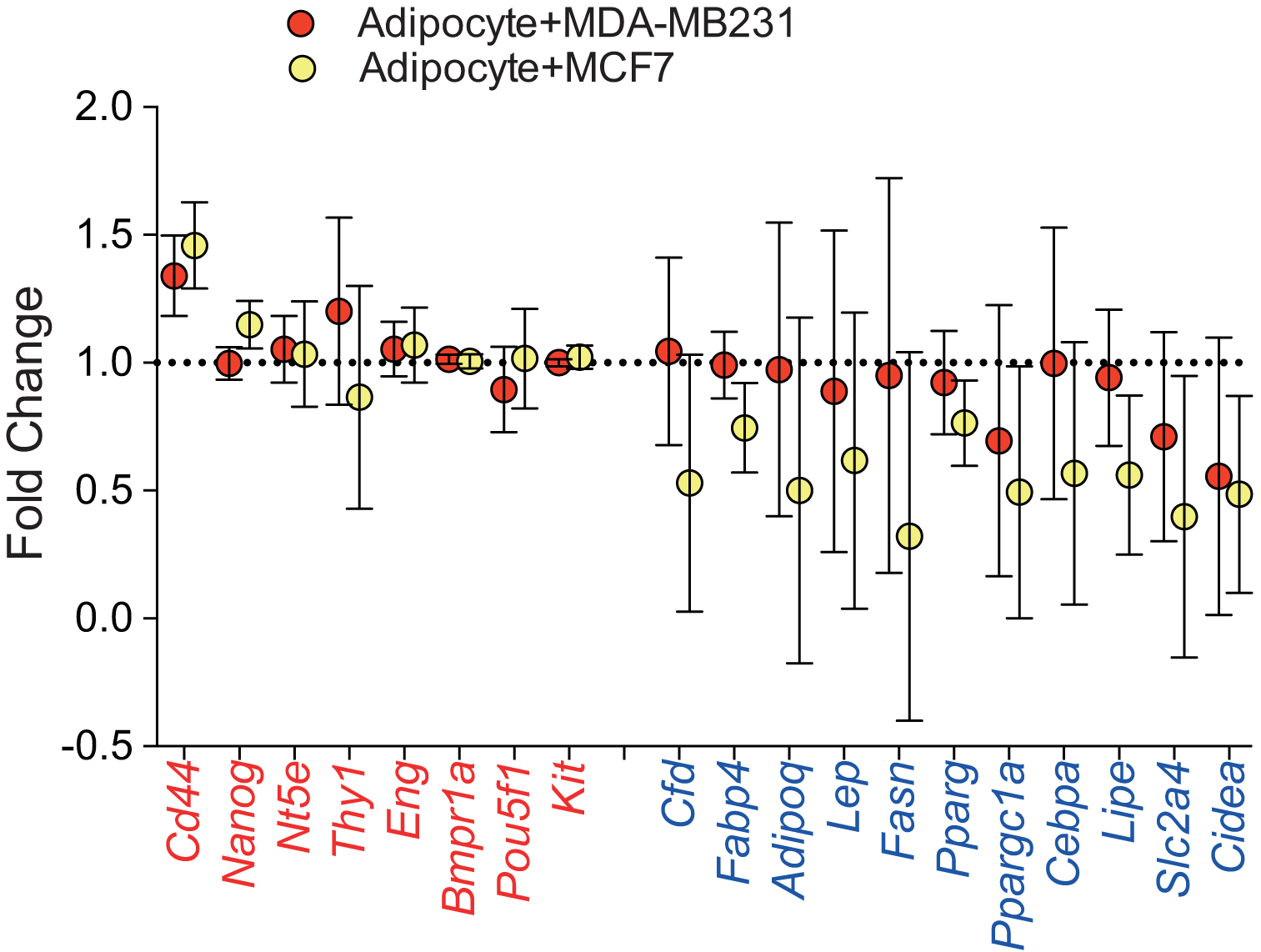

Supplement: S3 Fig — Genes in red and blue represent the genes expressed in mesenchymal stem cells and in differentiated adipocytes, respectively. (TIF) [file pone.0174126.s003.tif]
