# Supplementary material for: Transition into inflammatory cancer-associated adipocytes in breast cancer microenvironment requires microRNA regulatory mechanism
Source: PLoS One. 2017 Mar 23;12(3):e0174126. doi: 10.1371/journal.pone.0174126 (PMC5363867; doi:10.1371/journal.pone.0174126)
Supplement: S1 Table — The primers for PCR experiments for each gene is shown in the Table. (DOCX) [file pone.0174126.s004.docx]

**S1 Table. Primer sequences used in this study**

| *Cebpa* | Forward 5′- GCAAAGCCAAGAAGTCGGTG -3′ |
| --- | --- |
|  | Reverse 5′- CCTTGACCAAGGAGCTCTCA - 3′; |
| *Cebpb* | Forward 5′- GAAGACGGTGGACAAGCTGA -3′ |
|  | Reverse 5′- GCTTGAACAAGTTCCGCAGG -3′ |
| *Fabp4* | Forward 5′- GGATTTGGTCACCATCCGGT -3′ |
|  | Reverse 5′- CTCTTGTGGAAGTCACGCCT -3′ |
| *Pparg* | Forward 5′- AAGCCGTGCAAGAGATCACA - 3′ |
|  | Reverse 5′- ATCTTCTGGAGCACCTTGGC -3′. |
| *Il6* | Forward 5′- TCCAGTTGCCTTCTTGGGAC -3′ |
|  | Reverse 5′- GACAGGTCTGTTGGGAGTGG -3′; |
| *Bcl3* | Forward 5′- AACATAGCCGCTGTCTACCG -3′ |
|  | Reverse 5′- ATGTGGTGATGACAGCCAGG -3′ |
| *Ptx* | Forward 5′- CTGCAGTAGTGGCTGAGACC -3′ |
|  | Reverse 5′- TGAACAGCTTGTCCCACTCC -3′ |
| *Runx1* | Forward 5′- CCACAAGTTGCCACCTACCA -3′ |
|  | Reverse 5′- GCAGCTGCTCCAATTCACTG -3’; |
| *Il33* | Forward 5′- TGCATGAGACTCCGTTCTGG -3′ |
|  | Reverse 5′- TCCCGTGGATAGGCAGAGAA -3’ |
| *Gapdh* | Forward 5′- CATCTTCCAGGAGCGAGACC -3 |
|  | Reverse 5′- CTCGTGGTTCACACCCATCA -3′ |
